# Impaired Glucose Metabolism, Anti-Diabetes Medications, and Risk of Thyroid Cancer

**DOI:** 10.3390/cancers14030555

**Published:** 2022-01-22

**Authors:** Yevgeniya Kushchayeva, Sergiy Kushchayev, Kirk Jensen, Rebecca J. Brown

**Affiliations:** 1 Diabetes and Endocrinology Center, University of South Florida, Tampa, FL 33612, USA; 2 Department of Radiology, Moffitt Cancer Center, Tampa, FL 33612, USA; Sergiy.Kushchayev@moffitt.org; 3 F. Edward Hébert School of Medicine, Uniformed Services University, Bethesda, MD 20814, USA; kirk.jensen@usuhs.edu; 4 National Institute of Diabetes and Digestive and Kidney Diseases, National Institutes of Health, Bethesda, MD 20892, USA; brownrebecca@niddk.nih.gov

**Keywords:** thyroid abnormalities, insulin resistance, diabetes mellitus, anti-diabetic drugs, cancer risk

## Abstract

**Simple Summary:**

An epidemiologic link exists between obesity, insulin resistance, diabetes, and some cancers, such as breast cancer and colon cancer. The prevalence of obesity and diabetes is increasing, and additional epidemiologic data suggest that there may be a link between obesity and risk of thyroid abnormalities. Factors that may link obesity and diabetes with thyroid proliferative disorders include elevated circulating levels of insulin, increased body fat, high blood sugars, and exogenous insulin use. However, mechanisms underlying associations of obesity, diabetes, and thyroid proliferative disorders are not yet fully understood. The present manuscript reviews and summarizes current evidence of mechanisms and epidemiologic associations of obesity, insulin resistance, and use of anti-diabetes medications with benign and malignant proliferative disorders of the thyroid.

**Abstract:**

The prevalence of obesity is progressively increasing along with the potential high risk for insulin resistance and development of type 2 diabetes mellitus. Obesity is associated with increased risk of many malignancies, and hyperinsulinemia has been proposed to be a link between obesity and cancer development. The incidence of thyroid cancer is also increasing, making this cancer the most common endocrine malignancy. There is some evidence of associations between obesity, insulin resistance and/or diabetes with thyroid proliferative disorders, including thyroid cancer. However, the etiology of such an association has not been fully elucidated. The goal of the present work is to review the current knowledge on crosstalk between thyroid and glucose metabolic pathways and the effects of obesity, insulin resistance, diabetes, and anti-hyperglycemic medications on the risk of thyroid cancer development.

## 1. Introduction

Overweight and obesity are associated with increased risk for various malignancies, including liver, colorectal, and endometrial cancers [1,2,3,4,5]. Although less well established, obesity may also be a risk factor for thyroid cancer [6,7,8]. The incidence of thyroid cancer has risen in conjunction with the rise in prevalence of obesity, insulin resistance and type 2 diabetes, almost doubling since 2000 in the United States [9]. Although this is likely partly attributable to increased use of thyroid imaging [9], there are biological reasons to suspect an etiologic relationship between the rise in both obesity and thyroid cancer. Obesity-associated insulin resistance, hyperglycemia, and ectopic lipid deposition in non-adipose tissue have all been explored as possible contributors to tumorigenesis [10,11]. In this review, we discuss the data that has been compiled on associations between obesity, hyperinsulinemia, insulin resistance, diabetes, and anti-diabetes medications with thyroid cancer. We also explore potential molecular mechanisms, including hormone-receptor crosstalk and glucose pathways, which may explain the underlying relationships.

## 2. Obesity, Insulin Resistance, and Thyroid Cancer

### 2.1. Thyroid Cancer and Obesity

Epidemiologic data suggest a link between obesity and increased risk of thyroid abnormalities. An increase in body mass index (BMI) of 5 kg/m${}^{2}$ was shown to increase risk of thyroid cancer in men and women [1,7]. A pooled analysis of 12 case-control studies including 2473 patients with TC and 4323 controls showed that women with thyroid cancer had slightly higher weight and BMI versus controls [12]. A recent meta-analysis of 21 studies demonstrated that obesity (defined as BMI ≥ 30.0 kg/m${}^{2}$) was significantly associated with thyroid cancer risk as compared to normal weight [10]. No relationship was found between BMI/waist-to-hip ratio and thyroid cancer risk in patients without diabetes [13]. The risk of papillary thyroid cancer (PTC) in men with body fat > 27.9% and women with body fat > 37.8% was about 4 times higher as compared to that of subjects with body fat percentage in the lowest quartile [14]. However, despite the association between obesity and diagnosis of thyroid cancer, obesity has not been associated with aggressive histological thyroid cancer features, tumor recurrence, or disease persistence [7]. Of note, the association between obesity and thyroid cancer has only been demonstrated for cancers derived from thyroid epithelial cells such as PTC, follicular thyroid cancer (FTC), and anaplastic thyroid cancer (ATC). In medullary thyroid cancer (MTC), which is a cancer of neural cell crest origin, the association with obesity is reversed, with decreased risk of MTC occurrence [10]. These and additional studies are summarized in Table 1.

### 2.2. Obesity and Ectopic Thyroid Fat

The role of ectopic lipid in contributing to obesity-associated thyroid disease has not been well explored. Normal thyroid gland commonly contains a few adipocytes near the capsule and perivascular regions [15]. The presence of ectopic fat accumulation in follicular cells (thyroid steatosis) was demonstrated in patients with increased BMI after consuming a high fat diet [16]. However, there is no known relationship between ectopic lipid in the thyroid and increased risk of thyroid cancer.

### 2.3. Hyperinsulinemia and Thyroid Cancer

Hyperinsulinemia, as a consequence of insulin resistance, is associated with obesity and has been proposed as a link between obesity and cancer development [17,18,19,20], including differentiated thyroid cancer (DTC) [21,22,23]. An association of insulin resistance with increased thyroid volume and risk of benign and malignant thyroid lesions has been reported [22,23,24,25,26], especially PTC in young patients (≤44 years) [23]. Patients with signs of insulin resistance have a higher prevalence of thyroid nodules, larger size of thyroid nodules and increased thyroid volume [27,28,29]. Similarly, patients with goiter were shown to have higher insulin resistance (measured by homeostasis model of insulin resistance, HOMA-IR) and BMI [30]. Non-obese patients with extreme insulin resistance due to pathogenic variants in the insulin receptor (Rabson-Mendenhall syndrome) and high circulating insulin levels have been found to have thyromegaly [31], supporting insulin as a mediator of the link between common obesity and thyroid proliferation.

## 3. Growth Factors, Thyroid Proliferation, and Thyroid Cancer

Thyroid cells are slowly dividing cells, undergoing approximately five cell divisions in adulthood. Although thyroid volume does not increase after adolescence, the thyroid gland has the capacity to hypertrophy and proliferate in response to stimuli [32]. Thyroid growth is regulated by intrinsic systemic and locally produced factors including thyroid stimulating hormone (TSH), epidermal growth factor (EGF), transforming growth factors alpha and beta (TGF α and β), insulin-like growth factors 1 and 2 (IGF-1 and 2), fibroblast growth factor (FGF), hepatocyte growth factor (HGF), platelet-derived growth factor (PDGF), and insulin [33,34].

### 3.1. Mitogenic Actions of TSH

TSH is the major growth factor for thyroid cells and causes hyperplastic and mitogenic effects via multiple mechanisms. TSH controls thyrocyte growth and proliferation by binding to the TSH receptor, leading to activation of adenylate cyclase (AC), followed by increased cyclic adenosine monophosphate (cAMP) and protein kinase A (PKA). TSH binding to its receptor can also activate the phospholipase C (PLC) cascade and the receptortyrosine protein kinase (RTK) pathway [35]. The PLC and RTK pathways can also be activated by other growth factors such as EGF, TGF alpha, FGF, insulin, and IGF-1 [35].

TSH signaling is well recognized to work through PI3K/AKT and MAPK pathways. The PI3K/Akt cascade was shown to be the major proliferative signal for thyroid follicular cells in vivo in a mouse model [36]. In thyroid cells, the PI3K pathway can be activated by other factors such as insulin, IGF1, EGF, HGF [37]. AKT activation by TSH was also previously demonstrated [38]. Chronic TSH exposure causes AKT and MAPK-independent mTOR/S6K1/S6 axis activation and proliferation. TSH can also lead to S6K1 phosphorylation on Thr${}^{421}$/Ser${}^{424}$ independent of mTOR activation and independent of AKT or MAPK signaling [39]. Furthermore, TSH can bind to the TSHR and stimulate production of some growth factors such as FGF [40].

### 3.2. Insulin Acts as a Co-Mitogen with TSH via the Insulin Receptor (IR)

The mitogenic actions of TSH are limited in the absence of other growth factors, such as insulin or IGF-1. These co-mitogens are required for the mitogenic and protumorigenic actions of TSH to occur [32,41,42,43,44]. The co-mitogenic effects of TSH with a low physiological dose of insulin in vitro were demonstrated to occur via insulin receptor (IR) activation. By contrast, co-mitogenic effects of a high insulin concentration under TSH stimulation occurs via either the IR or through IGF1R signaling. In the absence of TSH, mitogenic effects of insulin are mainly via IGF1R [45].

The thyroid gland is not a classical insulin sensitive tissue such as liver, fat, or muscle [46], but insulin receptors are expressed in thyrocytes [24,47] and this expression is dependent on sustained stimulation by TSH via cAMP [45]. Differentiated cells express predominantly IR-B receptors, whereas cancer cells, especially poorly differentiated anaplastic or stem-like thyroid cancer cells, express predominantly IR-A receptors. IR-A receptors are typically present on fetal tissue and have high affinity with insulin and IGF-2 [24,48]. Changes in thyroid hormone status can also alter IR expression, upregulating IR in the hyperthyroid state and downregulating IR in the hypothyroid state [49].

Patients with insulin resistance and type 2 diabetes have increased circulating insulin, and hyperinsulinemia is considered to be one of the major contributors to increased risk of cancer. Insulin stimulates two main signaling pathways, including the IRS/PI3 kinase pathway, which is responsible for metabolic insulin actions, and the MAPK pathway, which controls growth and proliferation [50]. In patients with insulin resistance, such as in obesity and type 2 diabetes, it is thought that insulin signaling via the MAPK pathway is preserved, in contrast to impairment of the PI3K/AKT pathway, promoting more growth and proliferation effects.

### 3.3. Crosstalk between Insulin, Insulin like Growth Factors, and Their Receptors Contributes to Mitogenesis

IR and IGF-1 receptors (IGF-1R) play important roles in the induction and maintenance of cell proliferation, differentiation and phenotype, and are essential for carcinogenic transformation, tumor growth, and metastases. There is similarity between IGF1R and IR, including genomic organization (12 of 21 exons are identical), an 84% similarity in the tyrosine kinase domain of the β subunit, 48% homology in the cysteine-rich domains, 64–67% resemblance in the extracellular α subunit regions, and conservation of 15 out of 16 putative N-linked glycosylation sites [51]. However, each ligand has higher affinity to its cognate receptor. IGF-1 and IGF-2 bind to IR with an affinity 100–500-fold and 10–50-fold, respectively, lower than insulin [51]. IGF-1 has 1000-fold greater affinity to the IGF-1R than insulin [52].

Insulin signaling via the IR can stimulate mitogenic and anti-apoptotic effects similar to IGF-1R signaling, and IGF-2 may also activate the IR [53]. Hyperinsulinemia can stimulate IGF-1 receptors, resulting in overstimulation of MAPK pathways and promoting mitogenic effects [54,55,56,57]. Insulin and IGF-1 acting through IGF-1R induce protein synthesis and cell hypertrophy in human thyroid cells [41]. Tissue-specific knockout of the IR in cultured skeletal muscle, bone and thyroid tissue was shown to lead to overexpression/activation of IGF-1R in vitro and in vivo [47,58,59]. Moreover, significantly higher expression of tumor IGF-1R was found in patients with type 2 diabetes with some cancers (non-small cell lung cancer and colorectal cancer) [60,61]. Recently, thyromegaly was shown to be present in patients with homozygous insulin receptor pathogenic variants, as well as significantly increased prevalence of thyroid nodules in patients with extreme insulin resistance (Rabson-Mandenhall and lipodystrophy syndromes) [31].

Another growth factor, IGF-1, has been shown to be locally produced by stromal thyroid cells with higher expression in malignant tissue compared to normal. IGF-1 can activate IGF-1R and hybrid IR/IGF-1R receptors to a similar extent [53,62]. Crosstalk between the TSHR and IGF-1R has been demonstrated both in regulation of normal thyroid function in human thyrocytes in vitro, as well as in orbital fibroblasts responsible for the development of thyroid-induced ophthalmopathy [63,64].

Expression of IRs and IGF-1R is higher in thyroid cancer tissue in comparison to normal tissue. Analysis of IGF-1R expression in thyroid malignancies showed higher levels compared to normal thyroid tissues, with significantly higher expression in PTC and FTC than ATC and MTC [65]. Similarly, a tumor type-dependent trend was described for IR expression. Higher IR content was found in poorly differentiated and anaplastic TCs in contrast to well differentiated PTC [62], whereas higher expression of IGFRs was found in PTC in comparison to poorly differentiated/undifferentiated tumors with greatly overexpressed IRs (mainly IR-A) [53].

Hybrid insulin-IGF receptors are a different type of receptor that result from the random assembly of both receptors. These hybrid receptors have high affinity to bind IGF-1 but not insulin [66]. Both IR-A and IR-B can form hybrid receptors [67]. However, hybrid receptors containing IR-A bind IGF-1 and IGF-2, whereas hybrid receptors containing IR-B have affinity solely with IGF-1 [67]. Both IR and IGF-1R can be overexpressed in TC, especially in poorly differentiated TC, that can lead to hybrid receptor formation [68]. Similarly, a significantly higher presence of hybrid receptors was found in all thyroid malignancies in contrast to normal thyroid tissue, representing 60–70% of total IGF-1 binding sites [53,62].

IGF-2 is produced by malignant thyrocytes locally [53], and malignant transformation with activation of an autocrine loop involving IGF-2 and IR-A has been described [53]. Due to high homology of IGF-1 and IGF-2 to each other and to insulin, and involvement in cell proliferation, IGF-2 could also play a role in the development of thyroid nodules and/or thyromegaly. In insulin resistance, hyperinsulinemia can inhibit hepatic production of both IGF binding proteins (BP)-1 and 2, thus enhancing their bioavailability [69]. There is limited data about the effects of IGF-2 on thyroid proliferation. In the general population, no correlation has been observed between IGF-2 and thyroid gland size but IGF-2 positively correlated with thyroid hormone levels [70].

Recently, IGF-2BP3 overexpression was shown to promote cancer cell proliferation, invasion and transformation [71]. IGF-2BP3 overexpression was described in large nodules (mean 3.8 cm) with the follicular growth pattern typical for the follicular variant of PTC or noninvasive follicular thyroid tumor with papillary-like nuclear features [71].

## 4. Hyperglycemia, Diabetes, and Thyroid Cancer

Cancer and diabetes are associated more frequently than predicted by chance and have some similar risk factors [72]. Diabetes is associated with increased risk of certain cancers including endometrial, hepatic, pancreatic, colorectal, breast, and bladder cancers [72]. Patients with type 2 diabetes have been shown to have a higher prevalence of thyroid nodules in comparison to pre-diabetic and control groups [28]. Thyroid nodule size and volume positively correlated with HOMA-IR in males and females with type 2 diabetes [73].

Some studies have demonstrated increased risk of thyroid cancer in patients with diabetes [22,26,74,75,76] whereas others have not [13,75,77]. A recent meta-analysis on the association of type 2 diabetes with thyroid cancer risk showed a 20% increase in thyroid cancer risk among all subjects with type 2 diabetes and a 30% increase among women with type 2 diabetes [22]. The National Institutes of Health-AARP Diet and Health Study, including ∼500,000 participants, showed the absolute risk of thyroid cancer in patients with diabetes was 24.5/100,000 woman-years and 9.7/100,000 man-years in comparison to 16.0/100,000 woman-years and 8.8/100,000 man-years in the non-diabetic population [26]. Analysis of 16 cohort studies showed a significantly increased risk for thyroid cancer in women with diabetes but not men [76]. By contrast, the Women’s Health Initiative, a large prospective study of postmenopausal women ages 50–79 years, found no association of diabetes with thyroid cancer [13].

Factors that can link diabetes and thyroid cancer risk could include, but are not limited to, elevated circulating level of insulin, increased body fat, hyperglycemia, and exogenous insulin use. Hyperglycemia may link obesity with thyroid cancer in patients with overt diabetes. Warburg demonstrated that cancerous cells take up more glucose in comparison to normal cells, and predominantly depend on anaerobic glycolysis rather than oxidative phosphorylation for ATP production (termed the Warburg effect) [78]. This mechanism was proposed to support the biosynthetic requirements for uncontrolled cell proliferation by using increased glucose consumption as a carbon source for de novo generation of nucleotides, lipids, and proteins [79]. Though less efficient at generating ATP, the glucose metabolic rate via aerobic glycolysis is much higher than mitochondrial respiration, with lactate production that is 10–100 times faster [79]. The Warburg effect also serves as the physiological explanation for the increased uptake of ${}^{18}$F-FDG (fluorodeoxyglucose) by cancers, since most malignancies have an increased rate of glycolysis and glucose transport [80].

Hyperglycemia can affect cell growth and proliferation due to increased production of reactive oxygen species (ROS) and oxidative stress levels, which can lead to DNA mutations and may play a role in the initiation and progression of multistage carcinogenesis [80]. Experimental evidence suggests further roles for hyperglycemia in tumorigenesis and cancer progression. Hyperglycemia can: protect cancer cells from apoptosis; stimulate cell motility and invasiveness via epithelial-to-mesenchymal transition induction and ROS-mediated vascular destruction; regulate expression of proliferation-related genes, adhesion, and migration; stimulate expression of glucose transporters GLUT1 and GLUT3 in some cancers; and indirectly increase the levels of insulin/IGF-1 as well as inflammatory cytokines [80,81,82,83]. With respect to thyroid cancers, specifically, TSH was shown to stimulate the expression of the glucose transporter GLUT2 on β-cells, leading to increased glucose-stimulated insulin secretion in vivo and in vitro [84]. Multiple glucose transporters, including GLUT 1, 2, 3, 4, and 10, are expressed in the thyroid [85,86,87,88,89]. Increased expression of GLUTs has been observed in thyroid cancers [86,87,88], and increased membrane localization of GLUTs in advanced thyroid cancers [86,89]. This suggests that increased glucose transport may be a cause or consequence of thyroid cancer development.These and additional in vitro and in vivo studies are summarized in Table 2.

## 5. Diabetes Drugs and Thyroid Cancer

### 5.1. Sulfonylureas

Sulfonylureas, as well as glinides and insulin, are associated with increased risk of cancer [77]. Some studies demonstrated increased risk of cancer in patients taking sulfonylureas in comparison to those on metformin or other glucose lowering medications [90,91,92,93] but it is unclear if this effect is due to adverse effects of sulfonylureas or anti-proliferative effects of metformin [72]. First generation sulfonylureas were goitrogenic in animals and humans [94,95], and gliclazide, a second generation sulfonylurea, increased thyroid volume and decreased iodine uptake in the thyroid with no change in thyroid function [96].

### 5.2. Metformin

Metformin, an insulin sensitizer, is the most commonly used first line therapy in type 2 diabetes and has emerged as a potential anti-cancer drug. Metformin decreased overall cancer risk based on a large insurance claims database [97], and improved survival in some cancers, such as breast and colorectal [98,99]. Metformin reduces circulating insulin by improving hepatic insulin sensitivity and downregulating gluconeogenesis. One mechanism of metformin action is inhibition of mTORC1 by AMPK activation. mTORC1 is activated by glucose, amino acids, nutrients, growth factors, cellular energy, and mitogens, and stimulates anabolic reactions [100]. Furthermore, metformin inhibits oxidative phosphorylation and suppresses lipogenesis and malonyl-CoA synthesis, leading to increased fatty acid oxidation and reduced ATP production [101,102]. Thus, energy deficiency in cancer cells, secondary to metformin-induced ineffective AMPK signaling as the cell’s energy sensor, may lead to cancer cell death [103].

Metformin has been shown to have antimitogenic and antiproliferative effects on follicular and anaplastic thyroid cell lines and increased anti-cancer effects in combination with doxorubicin and cisplatin [104]. Many, but not all studies suggest beneficial associations of metformin with thyroid proliferation and tumorigenesis. Metformin reduced thyroid volume in patients with type 2 diabetes [105], likely due to direct inhibition of thyroid growth by activation of the AMPK/ mTOR pathway and antagonizing the growth-stimulatory effect of insulin by inhibition of the MAPK pathway [104]. In patients with insulin resistance, metformin with and without thyroid hormone therapy decreased the size of thyroid nodules by 30% and 55%, respectively, in comparison to placebo or thyroid hormone therapy alone [106]. In patients with type 2 diabetes, metformin reduced thyroid cancer risk [107] and was associated with higher thyroid cancer remission [108].

A large retrospective cohort study, based on the Korean National Health Insurance claim database, demonstrated significantly lower thyroid cancer risk (31% reduction) in metformin users vs. non-users of both sexes, with greater benefits at higher doses and with longer duration of use [109]. Similar thyroid cancer risk reduction (32%) in metformin users was demonstrated in the Taiwanese population [107]. Importantly, not all studies support a beneficial effect of insulin sensitizing drugs on thyroid cancer. No association of metformin treatment with thyroid cancer was shown in the Women’s Health Initiative [13]. Neither metformin nor pioglitazone or rosiglitazone altered thyroid cancer risk based on analysis of a Taiwanese insurance database [77].

### 5.3. Thiazolidinediones (TZD)

TZDs are selective agonists of peroxisome proliferator-activated receptor (PPAR) gamma which, when activated, alters gene transcription involved in glucose and lipid metabolism and energy balance [110]. Rosiglitazone induced apoptosis in PPAR gamma positive thyroid cancer cell lines and increased radioiodine uptake in dedifferentiated thyroid tumors [23]. Rosiglitazone also reversed the epithelial-mesenchymal transition and led to redifferentiation (increased expression of thyroglobulin, TSH receptors, sodium/iodide symporter (NIS) and thyroid peroxidase (TPO) mRNA) in ATC cell lines [111]. Troglitazone increased expression of the proapoptotic regulatory gene *c-myc* with inhibition of tumor growth and prevention of distant metastases in PPAR gamma positive PTC cell lines in vivo [112]. The PAX8/PPAR gamma fusion gene, which is created by balanced translocation between chromosomes 2 and 3, is involved in the tumorigenesis of follicular thyroid tumors. Pioglitazone was effective in reducing PAX8/PPAR gamma in a metastatic thyroid cancer model in vivo [113].

Human data from small groups of patients showed that TZDs increase radioactive iodine uptake in some patients, but this was not demonstrated by other authors [114,115,116,117,118]. Comparison of rosiglitazone users versus never-users showed no difference in incidence of thyroid cancer [119]. In addition, a trial of rosiglitazone treatment of 20 patients with advanced thyroid cancer did not show significant benefit [120]. However, epidemiologic data suggested that rosiglitazone was protective against thyroid cancer when duration of therapy was ≥14 months, or cumulative dose was ≥1800 mg, especially in patients ≥50 years old [119].

### 5.4. Glucagon-like Peptide 1 (GLP-1) Receptor Agonists

GLP-1 is a peptide hormone secreted by intestinal enteroendocrine cells that promotes insulin secretion in a glucose-dependent manner. Multiple actions of GLP-1 have been described, including increased β-cell mass, inhibition of glucagon secretion and gastric emptying, and neurotrophic effects on brain cells. GLP-1 receptor agonists bind to GLP-1 receptors (GLP-1R) on pancreatic β-cells and stimulate insulin secretion. Use of GLP-1 receptor agonists in rodents was shown to cause C-cell proliferation, increased calcitonin secretion and even development of C-cell carcinoma [121].There is no known association between GLP-1 receptor agonists and PTC [122]. With respect to risk of MTC with GLP-1 receptor agonists, data have been mixed regarding expression of GLP-1R in healthy human thyroid and human thyroid cancers [123,124]. Importantly, however, human data to date have not demonstrated convincing evidence of an increased risk of MTC with use of these drugs [125,126,127]. However, since there is still controversy regarding the possibility of GLP-1 agonists increasing risk of some malignancies in humans, including MTC, a personal or family history of MTC or MEN2 are contraindications for GLP-1 based therapy.

### 5.5. Dipeptidyl Peptidase 4 (DPP4) Inhibitors

DPP4 (also known as CD26) is a member of the dipeptidyl peptidase family that can cleave N-terminal dipeptides and degrade GLP-1 [128]. DPP4 inhibitors thus prolong the actions of GLP-1 described above, resulting in better blood glucose control [129]. A recent meta-analysis of 72 RCTs that compared patients on DPP4 inhibitors with untreated patients did not show an association of DPP4 with cancer development, including thyroid cancer [130]. However, there is reason to consider DPP4 as a potential cancer target. DPP4 is upregulated in a variety of tumors including T-cell malignancies, B-cell chronic lymphocytic leukemia, lung, esophageal, and prostate cancers [128]. DPP4 expression occurs in both benign and malignant thyroid tissue, and is related to the proliferative activity of follicular cells [131]. DPP4 expression is increased in PTC [132], particularly in patients with more advanced disease [133]. DPP4 silencing or inhibition suppressed PTC colony formation, cell migration, and invasion in vitro and inhibited tumor growth in vivo [133].

### 5.6. Meglitinides

Meglitinides inhibit β-cell potassium channels, thus stimulating insulin secretion. Meglitinides were shown to be associated with risk of cancers [97]. However, no clear evidence of increased risk of thyroid cancer has been documented to date.

### 5.7. Sodium-Glucose Cotransporter 2 (SGLT2) Inhibitors

SGLT2 inhibitors affect renal glucose reabsorption via inhibition of SGLT2, thus increasing urinary glucose excretion. A systematic review and meta-analysis of 46 RCTs did not show significant increase in overall cancer risk [134]. Canagliflozin might have a protective effect to decrease risk of GI cancers compared to placebo. However, there was evidence that SGLT2 inhibitors are associated with increased risk of overall cancer and bladder cancer in patients with BMI > 30 kg/m${}^{2}$ that was not demonstrated in normal weight participants or participants with overweight [134]. Currently, there is no evidence of association between SGLT2 inhibitor use and thyroid cancer.

### 5.8. Leptin

Leptin is produced by adipocytes in proportion to fat stores, and thus represents a possible link between obesity and cancer. Potential proliferative effects of leptin occur via its stimulation of MAPK, JAK/STAT3, and PI3K/AKT, promoting cell proliferation and differentiation. Leptin can also increase expression of cell cycle markers (Cyclin D1, CDK2, and c-Myc) leading to proliferation. Patients with PTC have higher leptin levels compared to healthy controls [135] independent of BMI [136]. Furthermore, leptin and its receptor were expressed in 37% and 51% of PTC tissue and lymph node metastases, respectively, vs. non-tumor tissue, and expression was associated with greater neoplasm size and lymph node metastases [137]. However, administration of metreleptin, a recombinant analog of human leptin, to patients with extreme insulin resistance, including Rabson-Mendenhall syndrome and lipodystrophy, did not show increased risk of PTC compared to patients not treated with metreleptin [31].

### 5.9. Pramlintide

Pramlintide is a synthetic analog of amylin that is co-secreted with insulin by pancreatic β-cells. Pramlintide had anticancer effects in thymic lymphomas and colorectal cell lines via p53-dependent effects [138,139]. Pramlintide also showed synergistic benefits with chemotherapeutic agents [138]. Currently, there is no known association of pramlintide with thyroid cancer.

### 5.10. Insulin

Hyperinsulinemia may be a key link between obesity and increased risk of cancer [97]. Insulin use was associated with increased risk of cancer among all cumulative dosage and duration categories based on analysis of 108,920 patients with newly diagnosed diabetes [97]. As discussed above, supraphysiological concentrations of insulin can bind to the IGF-1Rs and mediate mitogenic effects [140]. Potential oncogenic effects of recombinant analogs of human insulin used for treatment of diabetes may relate to their differing binding affinities with the insulin vs. IGF-1 receptors [140]. Mitogenic potency of insulin analogs is correlated with IGF-1R affinity in most cases [140]. Two short-acting forms of insulin (aspart and lispro) and a long-acting insulin (glargine) have similar binding affinity with the IR as compared to human insulin, whereas the long-acting insulin analog detemir has less potency. By contrast, compared to human insulin, binding to the IGF-1R was similar for aspart, 1.5 times higher for lispro, 6.5 times higher for glargine and more that 5-fold lower for detemir.

In vitro studies of colorectal, prostate, and breast cancer lines showed that glargine, detemir, lispro, and aspart all stimulated cell proliferation more than regular human insulin [141]. Long-acting insulins have been shown to prevent apoptosis in colorectal cancer cell lines, resembling the effect of IGF-1. Insulin glargine stimulated phosphorylation of both IR and IGF-1R, in contrast to detemir, which only induced phosphorylation of IR but not IGF-1R in colorectal cells. Based on in vivo data, therapeutic doses of glargine do not cause thyroid cell proliferation [142]. Detemir had much lower capacity to stimulate AKT phosphorylation in comparison to glargine [141]. Despite increased stimulation of the IGF-1R by glargine, epidemiologic data on the association of glargine with cancer has been mixed. A meta-analysis of the effect of insulin exposure as well as type of insulin (glargine vs. non-glargine insulin) showed that the risk of developing cancer is dependent on cancer type [143]. Insulin users (including all types of insulin) versus non-insulin users had increased risk for pancreas, liver, kidney, stomach, and respiratory cancer, but decreased risk for prostate cancer. Use of glargine in contrast to non-glargine insulin was associated with increased risk for breast cancer and decreased risk for colon cancer. No increased risk of cancer with glargine vs. insulin NPH was seen [144,145]. To date, two studies have found no association between risk of thyroid cancer and exogenous insulin use [13,146] while one study showed a positive association of insulin use with thyroid cancer in women only [147].

## 6. Conclusions

**Figure 1:**
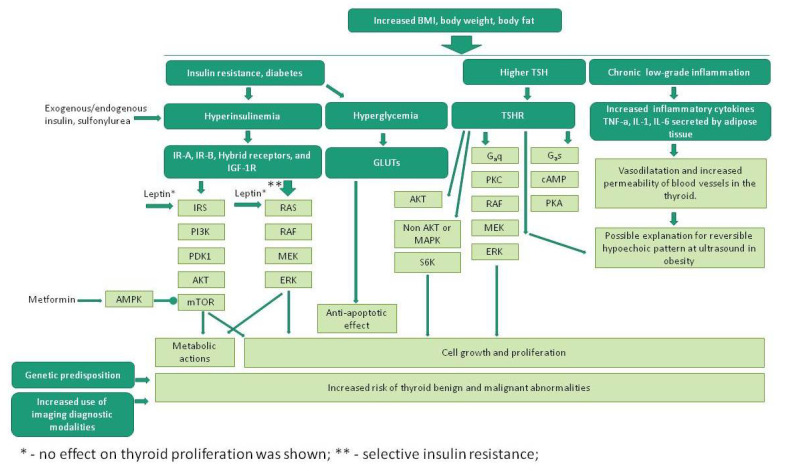
Effects of obesity, insulin resistance, DM on thyroid abnormalities.

There are complex relationships between obesity, chronic inflammation, hyperglycemia, hyperinsulinemia, genetic redisposition, crosstalk between proliferative pathways, and a variety of anti-diabetes medications with possible unknown off-target effects that might affect thyroid cancer risk Figure 1. Based on current evidence, it is not certain if insulin resistance or hyperglycemia increase risk of thyroid cancer. However, cur-rent evidence supporting associations of obesity, insulin resistance, and diabetes with thyromeg-aly, thyroid nodules and likely with thyroid cancer (at least in women), suggests that early screening for thyroid abnormalities in these patients may be warranted. Further investigations are required to confirm or refute potential associations of these risk factors with thyroid cancer in long-term prospective studies.

**Table 1:** Effects of glucose abnormalities on thyroid growth and proliferation in patients.

Risk Factor	Thyroid Abnormality	Literature Evidence	Reference
**Obesity, IR Hyperinsulinemia**	**Thyromegaly (TV by TUS)**	Obesity + IR vs. obesity without IR: TV 17 ± 3 vs. 14 ± 3 mL (*p* < 0.05) Normal weight + IR vs. normal weight without IR: TV 16 ± 2 vs. 12 ± 2 mL (*p* < 0.05) No difference between normal weight + IR and obesity + IR	[29]
		Class III obesity vs. control: TV 9 ± 2 vs. 16 ± 10 mL in men only (*p* < 0.05), positive correlation of TV with HOMA-IR	[27]
		Among patients with extreme insulin resistance: INSR^−/−^ vs. INSR^+/−^ vs. LD: TV 10 ± 5 vs. 4 ± 2 vs. 6 ± 3 mL (*p* < 0.05); fasting insulin 626 ± 390 vs. 136 ± 124 vs. 64 ± 121 μU/mL (*p* < 0.05)	[31]
**Pre-DM or DM**	**Thyromegaly (TV by TUS)**	Pre-DM and type 2 DM in a mild-to-moderate iodine deficient area: Pre-DM vs. DM vs. control: TV 18 ± 9 vs. 20 ± 8 vs. 11 ± 4 mL (*p* < 0.0001)	[28]
		DM vs. control: TV 12 ± 5 vs. 7 ± 2 mL for males; 10 ± 6 vs. 7 ± 3 mL for females (*p* < 0.001 for both)	[148]
		Type 1 DM vs. control: TV 17 vs. 14 mL (*p* < 0.05) Type 2 DM vs. control: TV 21 vs. 14 mL (*p* < 0.001)	[149]
		Type 1 DM vs. control: Goiter prevalence 29% vs. 36% (*p* < 0.05); no difference in TV	[150]
**Obesity, IRHyperinsulinemia**	**Thyroid nodules by TUS**	Obesity + IR vs. obesity without IR: Thyroid nodule prevalence 50 vs. 24% (*p* < 0.005) Normal weight + IR vs. normal weight without IR: Thyroid nodule prevalence 61 vs. 16% (*p* < 0.001)	[29]
		Observational associations: Obesity (BMI 30–40 kg/m^2^) vs. lean: odds of TN 1.38-fold (95% CI: 1.17–1.62) Each 1 SD (4.8 kg/m^2^) increase in BMI corresponded to 1.15 (95% CI: 1.08–1.22) higher odds of TN Mendelian randomization: no evidence that BMI can cause TN	[151]
**pre-DM or DM**	**Thyroid nodules by TUS**	Pre-DM and type 2 DM in a mild-to-moderate iodine deficient area: Pre-DM vs. DM vs. control: Thyroid nodule prevalence 51 vs. 62 vs. 24% (*p* < 0.0001)	[28]
		Type 1 DM vs. control: no difference in Thyroid nodule prevalence Type 2 DM vs. control: Thyroid nodule prevalence 48 vs. 28% (*p* < 0.05)	[149]
		Type 1 DM vs. control: Thyroid nodule prevalence 11 vs. 19% (*p* < 0.05)	[150]
		Observational associations: No association of Type 2 DM with thyroid nodules	[151]
**Obesity, IR,Hyperinsulinemia**	**Thyroid cancer**	Observational associations: Obesity was trending toward higher odds of thyroid cancer (OR 1.34; 95% CI: 0.99–1.84) but per unit higher BMI was not associated with thyroid cancer. Mendelian randomization: no causal link for obesity with thyroid cancer was found.	[151]
		IR present in 70% of patients with thyroid cancer and BMI > 25 vs. 20% in patients with BMI > 25 and without thyroid cancer	[152]
		BMI positively correlated with risk of thyroid cancer in both genders; Risk of thyroid cancer rose with increasing BMI	[153]
		Higher risk of thyroid cancer for participants with a higher BSA, height, weight, or body fat percent (women only)	[154]
		BMI was associated with DTC risk in women only	[12]
		Thyroid cancer increased with increasing BMI (Relative risk of thyroid cancer per unit increase in BMI 1.03 (95% CI: 1.00–1.05) in men and 1.02 (95% CI: 1.01–1.03) in women)	[155]
		BMI > 35.0 vs. normal BMI: Hazard ratio of TC in women 1.74 (95% CI: 1.03, 2.94) with no difference in men	[156]
		No associations between incidence of thyroid cancer and either weight or BMI. However, the proportion of participants with BMI ≥30 was only 3%.	[157]
		BMI and body fat percentage significantly associated with increased risk of PTC. Odd ratios of PTC for overweight and obese subjects (vs. lean) 1.72 [CI 1.48–2.00] and 4.17 [CI 3.41–5.10]. Odd ratios of PTC for highest quartile vs. lowest quartile of body fat percent 3.83 [CI 2.85–5.15] in women and 4.05 [CI 2.67–6.15] in men.	[14]
**Obesity, IR,Hyperinsulinemia**	**Changes depends on type of thyroid cancer**	With increasing BMI, the relative risk of FTC increased more than the risk of PTC. ATC has a strong positive association with BMI (in men only).Risk of MTC decreases with increasing BMI (in females only). The relative risk of MTC per 1 kg/m^2^ increase in BMI was 0.94 (95% CI: 0.85–1.04) in men and 0.91 (95% CI: 0.86–0.97) in women.	[155]
		IR is present in 56% of patients with PTC and 25% of patients with FTC	[152]
		Risk of PTC increases with a 5% increase in body fat percentage (Odds ratio 1.54, CI: 1.45–1.64) and with 5 kg/m^2^ increase in BMI (Odds ratio 1.77, CI: 1.64–1.91).	[14]
**pre-DM or DM**	**Thyroid cancer**	Individuals in the highest vs. lowest quartile of genetic risk of type 2 DM had higher odds of thyroid cancer (Odds ratio 1.45; CI: 1.11–1.90).	[151]
		DM was associated with increased risk of thyroid cancer in women > 60 years of age (RR 1.26; 95% CI 1.03–1.54)	[76]
		No association was observed between Type 2 DM and thyroid cancer (Hazard ratio 1.09; 95% CI: 0.79–1.52)	[13]
		Type 2 DM type 2 was associated with 1.34-fold (95% CI: 1.11–1.63) increased risk of thyroid cancer overall, with a 1.38-fold (95% CI: 1.13–1.67) increased risk in women but not in men (relative risk 1.11, 95% CI: 0.80–1.53)	[22]
		DM was associated with risk of thyroid cancer in women (Hazard ratio 1.46, 95% CI: 1.01–2.10). but not men (Hazard ratio 1.04, 95% CI: 0.69–1.58)	[26]
		No increased risk for thyroid cancer was observed in patients with DM (however, very few thyroid cancer cases existed in this study). Relative risk was 1.0 (95% CI: 0.6–1.8) for women and 1.3 (95% CI: 0.5–2.8) for men.	[158]
		No increased risk for thyroid cancer was observed in patients with DM (however, very few thyroid cancer cases existed in this study). Relative risk was 1.3 (95% CI: 0.6–2.3) for women, 1.2 (95% CI: 0.7–1.8) for men.	[159]
		No increased risk for thyroid cancer was observed in women with DM (Hazard ratio 1.74 [95% CI: 0.41–7.29])	[160]
		No increased risk of thyroid cancer was observed in patients with DM. Hazard ratio was 1.46 (95% CI: 0.83–2.56) in men. Hazard ratio was 0.83 (95% CI: 00.28–2.51) in women.	[161]
		Neither DM (Odds ratio 0.75, 95% CI: 0.21–2.73), nor DM duration were significantly associated with thyroid cancer.	[162]
**pre-DM or DM**	**Changes depends on type of thyroid cancer**	Women with DM had somewhat higher risk of FTC (Hazard ratio 1.92; 95% CI: 0.86–4.27) than PTC (Hazard ratio 1.25; 95% CI: 0.80–1.97)	[26]
**Summary**		1. There is a positive association of BMI, IR, and type 2 DM with thyroid size. 2. There is a positive association of BMI, IR and DM with thyroid nodules. 3. There is a likely increased risk of thyroid cancer with elevated BMI and DM in women only. More studies are needed. 4. There is insufficient data relating MTC and ATC with insulin or glucose abnormalities to draw conclusions.	

DTC—Differentiated thyroid cancer; PTC—papillary thyroid cancer, FTC—follicular thyroid cancer, MTC—medullary thyroid cancer, ATC—anaplastic thy-roid cancer, IR—insulin resistance, TV—thyroid volume, TN—thyroid nodules, TUS—thyroid ultrasound, LD—lipodystrophy; INSR—insulin receptor, −/− homozygous mutation, +/− heterozygous mutation, SD—standard deviation; SIR—Standardized incidence ratio, DM—diabetes mellitus.

**Table 2:** Effects of glucose abnormalities, insulin, and metformin on thyroid growth and proliferation in vitro and in vivo.

	Factor	Cells/Animals	Effect	Reference
**In vitro**	Human Insulin (HI) and Glargine Insulin (GI)	FRTL-5 (Follicular ratthyroid cells) PTC1 (human PTC cell line)	FRTL-5 cells: - Promotion of proliferation in a time- and dose-dependent manner; HI > GI - Activation of IGF-1R and IR in FRTL-5 cells - HI primarily acts on IR and GI mainly acts on IGF-1 at high insulin concentrations - High dose of GI induces more Akt phosphorylation than HI - No increase in Erk1/2 phosphorylation PTC-1 cells: - Promotion of proliferation - Higher mitogenic activity of GI vs. HI at high concentrations - Increase in cell migration; GI > HI - Both insulins activated IGF-1R and IR-High dose of GI induces more Akt phosphorylation than HI - Increase in Erk1/2 phosphorylation, HI > GI at high insulin doses	[163]
**In vitro**	Insulin: HI and GI	FRTL-5 (Follicular ratthyroid cells); FTC-133 (human FTC cancer cell line)	FRTL-5 cells: increased proliferation with both insulins FTC-133 cancer cells: no increase in cell proliferation	[164]
**In vivo** **In vitro**	Hyperglycemia Type 1 DM	Wistar female rats PCCL3 cell (thyroid epithelial cell line from adult Fisher rats)	- High glucose level caused increase in extra- and intracellular H${}_{2}$O${}_{2}$ pro-duction in thyrocytes through activation of the PKC pathway. - Upregulation of DUOX1 and NOX4 via activation of PKC and reduced TPO mRNA levels. - Insulin in the absence of TSH increased production of H${}_{2}$O${}_{2}$ more than hyperglycemia alone.	[165]
**In vitro**	Metformin	HTh74, C643, and SW1736 (human ATC cell lines) FTC133 (human FTC cell line)	- Inhibition of proliferation, cell cycle arrest and induction of apoptosis - Additive antiproliferative effect with doxorubicin or cisplatin - Abolished insulin-mediated cell growth of ATC cells - Dose-dependent decrease in ERK phosphorylation, including abolishing insulin-mediated ERK upregulation.	[104]
**In vitro**	Metformin	FTC-133 (human FTC cell line), K1E7 (subclone of K1 cell line from human PTC), RO82-W-1 (human FTC cell line), 8305C (human ATC cell line), TT (human MTC cell line), Nthy-ori 3-1 (human normal thyroid follicular cells)	- Inhibition of cell proliferation, colony formation, and cell migration - No effect on DNA repair - induction of cell cycle arrest and apoptosis	[166]
**In vivo**	High fat diet (HFD)-induced obesity	ThrbPV/PVPten ± mice (spontaneously develops metastatic FTC; animals harbor a mutated thyroid hormone receptor-β and haploinsufficiency of Pten)	HFD vs. low fat diet (LFD): - Stimulation of thyroid tumor growth - Increase in Ki-67 positive cells and protein abundance of p-Rb and cyclin D1 - No difference in activation of MAPK and PI3K signaling pathways - Promotion of anaplastic change in thyroid cancers - Increased expression of Mcl1, Bcl2l1, Ccnd1, and Vegfa (STAT3 target genes) and activation of leptin-JAK2-STAT3 signaling as one pathway that mediates obesity-induced aggressive tumor progression.	[167]
**In vivo**	Insulin:HI and GI	Female Wistar rats	- Dose-dependent effect on Akt and ERK1/2 phosphorylation, GI > HI - Higher and longer Akt and ERK1/2 phosphorylation after GI vs. HI - Dose-dependent increase in Ki-67 positive cells(HI vs. GI at highest doses) - High doses of HI primarily affect IR phosphorylation - High doses of GI primarily affect IGF-1R phosphorylation	[142]
**In vivo**	Metformin effect in obese mice	ThrbPV/PVPten± mice (spontaneously develops metastatic FTC; animals harbor a mutated thyroid hormone receptor-β and haplodeficiency of Pten	HFD vs. LFD: - Higher thyroid weight in HFD animals - Metformin delayed thyroid cancer progression by reducing in capsular invasion, abrogating development of vascular invasion and anaplasia, reducing p-STAT3 signals in thyroid tumor cells in animals on HFD, and decreasing extent of epithelial-mesenchymal-transition by vimentin inhibition.	[168]
**In vivo**	HFDMetformin	Female albino rats	HFD altered thyroid morphology, including thyroid follicles of varying diameters, excessive amount of colloid, vacuolated cytoplasm and disrupted basement membrane, irregular shrunken nuclei with dense chromatin, loss of apical microvilli, an apparent decrease in the number of ribosomes and secretory granules in some cells. Decrease in height of follicular epithelial cells. Metformin ameliorated effects of HFD on thyroid morphologyThyroid follicular cells of rats on HFD and metformin demonstrated near-normal structure	[169]

HI—Human insulin; GI—glargine insulin; IR—insulin receptors; IGF-1R—insulin like growth factor 1 receptor; HFD—high fat diet; LFD—low fat diet, FTC—follicular thyroid cancer, PTC—papillary thyroid cancer, MTC—medullary thyroid cancer.

## Data Availability

Not applicable.
